# Impact of a booster dose on SARS-CoV2 mRNA vaccine-specific humoral-, B- and T cell immunity in pediatric stem cell transplant recipients

**DOI:** 10.3389/fimmu.2023.1239519

**Published:** 2023-10-24

**Authors:** Linda Marie Laura Thole, Laura Tóth, Vanessa Proß, Janine Siegle, Carolin Stahl, Georg Hermsdorf, Annette Knabe, Annika Winkler, Eva Schrezenmeier, Carolin Ludwig, Cornelia Eckert, Angelika Eggert, Hubert Schrezenmeier, Arne Sattler, Johannes H. Schulte, Katja Kotsch

**Affiliations:** ^1^ Department of General and Visceral Surgery, Charite-Universitätsmedizin Berlin, Corporate Member of Freie Universität Berlin and Humboldt-Universität zu Berlin, Berlin, Germany; ^2^ Department of Pediatric Oncology and Hematology, Charite-Universitätsmedizin Berlin, Corporate Member of Freie Universität Berlin and Humboldt-Universität zu Berlin, Berlin, Germany; ^3^ Department of Nephrology and Medical Intensive Care, Charite-Universitätsmedizin Berlin, Corporate Member of Freie Universität Berlin and Humboldt-Universität zu Berlin, Berlin, Germany; ^4^ Berlin Institute of Health at Charité-Universitätsmedizin Berlin, Berlin Institute of Health (BIH) Academy, Clinician Scientist Program Universitätsmedizin Berlin, Berlin, Germany; ^5^ Institute of Transfusion Medicine, Ulm University, Ulm, Germany; ^6^ Institute for Clinical Transfusion Medicine and Immunogenetics, German Red Cross Blood Transfusion Service Baden-Württemberg – Hessen and University Hospital Ulm, Ulm, Germany; ^7^ Department of Pediatric Hematology and Oncology, University Children’s Hospital, Eberhard Karls University Tuebingen, Tuebingen, Germany

**Keywords:** SARS-CoV2, mRNA vaccination, stem cell transplantation, pediatrics, cellular immunity, humoral immunity, antigen-specific immunity

## Abstract

Stem cell transplant recipients (SCTR) are imperiled to increased risks after SARS-CoV2 infection, supporting the need for effective vaccination strategies for this vulnerable group. With respect to pediatric patients, data on immunogenicity of SARS-CoV2 mRNA-based vaccination is limited. We therefore comprehensively examined specific humoral, B- and T cell responses in a cohort of 2-19 year old SCTR after the second and third vaccine dose. Only after booster vaccination, transplant recipients reached similar levels of vaccine-specific IgG, IgA and neutralizing antibodies against omicron variant as age-matched controls. Although frequencies of SARS-CoV2 specific B cells increased after the third dose, they were still fourfold reduced in patients compared to controls. Overall, the majority of individuals enrolled mounted SARS-CoV2 Spike protein-specific CD4^+^ T helper cell responses with patients showing significantly higher portions than controls after the third dose. With respect to functionality, however, SCTR were characterized by reduced frequencies of specific interferon gamma producing CD4^+^ T cells, along with an increase in IL-2 producers. In summary, our data identify distinct quantitative and qualitative impairments within the SARS-CoV2 vaccination specific B- and CD4^+^ T cell compartments. More importantly, humoral analyses highlight the need for a booster vaccination of SCTR particularly for development of neutralizing antibodies.

## Introduction

Stem cell transplantation (SCT) is the treatment of choice for patients suffering from a plethora of malignant and non-malignant diseases. Early prospective studies on adults after autologous or allogenic SCT identified a strongly elevated risk to develop severe corona virus disease-19 (COVID19) with more than 20 percent of infected individuals being admitted to ICU, associated with poor overall survival rates (1). Since these observations had been validated in large meta-analyses (2), they supported the need for effective SARS-CoV2 vaccination strategies for this vulnerable patient group. Overall, immunogenicity of standard two-dose, mRNA-based vaccination was found impaired compared to healthy controls in both autologous and allogeneic adult SCT settings, with overall serological responder rates around 75% (3, 4). Whereas primary disease and intensity of conditioning had a minor impact on humoral responses in adult allogeneic transplant recipients, vaccination outcome was dependent on patient age, graft-versus-host-disease (GvHD), associated immunosuppressive medication and time since transplantation (4, 5). The latter also applied to autologous SCTR where B cell depleting therapies strongly impair development of humoral immunity (6). Administration of a booster dose significantly increased anti-SARS-CoV2 receptor binding domain (RBD)-specific IgG levels in the allo-setting (7) and substantially raised serological responder rates in autologous SCTR (8). Information on vaccine-induced T cell immunity in SCTR is often based on indirect assays, including detection of secreted IFNγ after peptide stimulation by ELISPOT or ELISA (9, 10), impeding a comprehensive analysis of specific T cell quantity and quality. Additional limitations in some reports include the absence of a matched control group (11); it therefore remains largely unaddressed whether and how SARS-CoV2-vaccination-specific T cell immunity is altered in stem cell transplant recipients, particularly in pediatric cohorts.

Limited data available on SARS-CoV2 infection in pediatric SCTR indicates lower COVID19-associated risks than determined for adults; however, around 10% of young patients required supplemental oxygen or mechanical ventilation and up to 8% died from infection (12, 13), thereby by far exceeding statistics determined for the general population (14). With respect to vaccination outcomes in pediatric patients, the report by Matkowska-Kocjan suggests similar serological responder rates amongst patients and healthy controls (15). However, previous SARS-CoV2 infection in the majority of individuals enrolled did not allow to assess the sole contribution of vaccination to overall responses. To provide comprehensive data on mRNA vaccine induced humoral and cellular responses in pediatric SCTR aged 2-19 years, we conducted an observational study where vaccine-specific IgG, IgA and Omicron variant neutralizing capacity was assessed in concert with extensive quantification and functional characterization of spike protein-specific B- and T cells. Our results strongly support the recommendation for a booster vaccination and identify both quantitative and functional impairments within anti-viral cellular immunity.

## Results

### Impact of SARS-CoV2 booster vaccination on specific humoral immunity in SCTR

**Figure 1:**
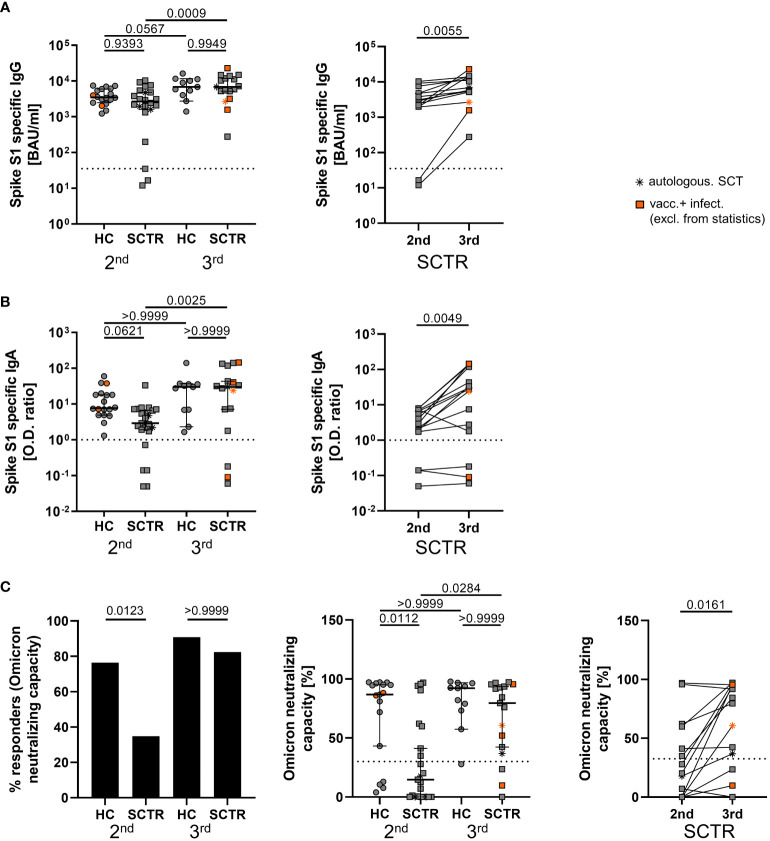
Assessment of SARS-CoV2-specific humoral responses. Humoral SARS-CoV2-specific immunity was assessed in healthy controls (HC) and stem cell transplant recipients (SCTR) after the second and third vaccine dose based on Spike protein S1 domain specific IgG **(A)** and IgA **(B)** by ELISA (left panels, respectively). For a limited number of patients, pairwise comparisons were conducted after the second and third dose (right panels, respectively). **(C)** Responder rates of individuals with Omicron variant neutralizing capacity above threshold (left), quantification of neutralizing capacity in percent (middle) and pairwise analysis (right) as determined by ELISA. Thresholds defining a positive response are indicated by dotted lines. Graphs show medians ± 95% CI. Patients after autologous SCT are marked as indicated. Vaccinated plus infected individuals are marked in orange; they were excluded from statistical analysis.

Humoral immunity was assessed by ELISA in pediatric SCTR and age-matched healthy controls approximately 5 weeks after the second and third vaccine dose. Spike S1 specific IgG levels were not substantially different between both groups at either time point. However, whereas quantities in healthy individuals only slightly increased after the third dose, SCTR significantly benefited from the booster as mirrored by more than doubled IgG levels (**Figure 1A**, left). This finding was also applicable to patients in a pairwise comparison after second vs. third dose where fewer individuals were included due to paired sample availability (**Figure 1A**, right). Similar results were obtained for specific IgA levels (**Figure 1B**). After the second dose, significantly fewer patients than controls showed Omicron neutralizing capacity above threshold. Importantly, this feature normalized after the third dose, resulting in similar portions of responders in healthy individuals and patients (**Figure 1C**, left) as well as percentage of neutralizing capacity (**Figure 1C**, middle). Neutralization capacity also increased after the third dose in a pairwise comparison in SCTR (**Figure 1C**, right). Neutralizing capacity levels did neither correlate with stem cell donor age nor time since transplantation (**Supplemental Figure 1A**). Whereas neutralizing capacity levels positively correlated with those of specific IgG except for HC after the 2nd dose (**Supplemental Figure 1B**), such associations were not noted for IgA (**Supplemental Figure 1C**). Datasets from the few individuals acquiring a SARS-CoV2 infection during the study period were included in all figures for gross comparison. However, as also mentioned in the respective figures and legends, they were excluded from statistics.

### Features of vaccine-specific B cell responses

**Figure 2:**
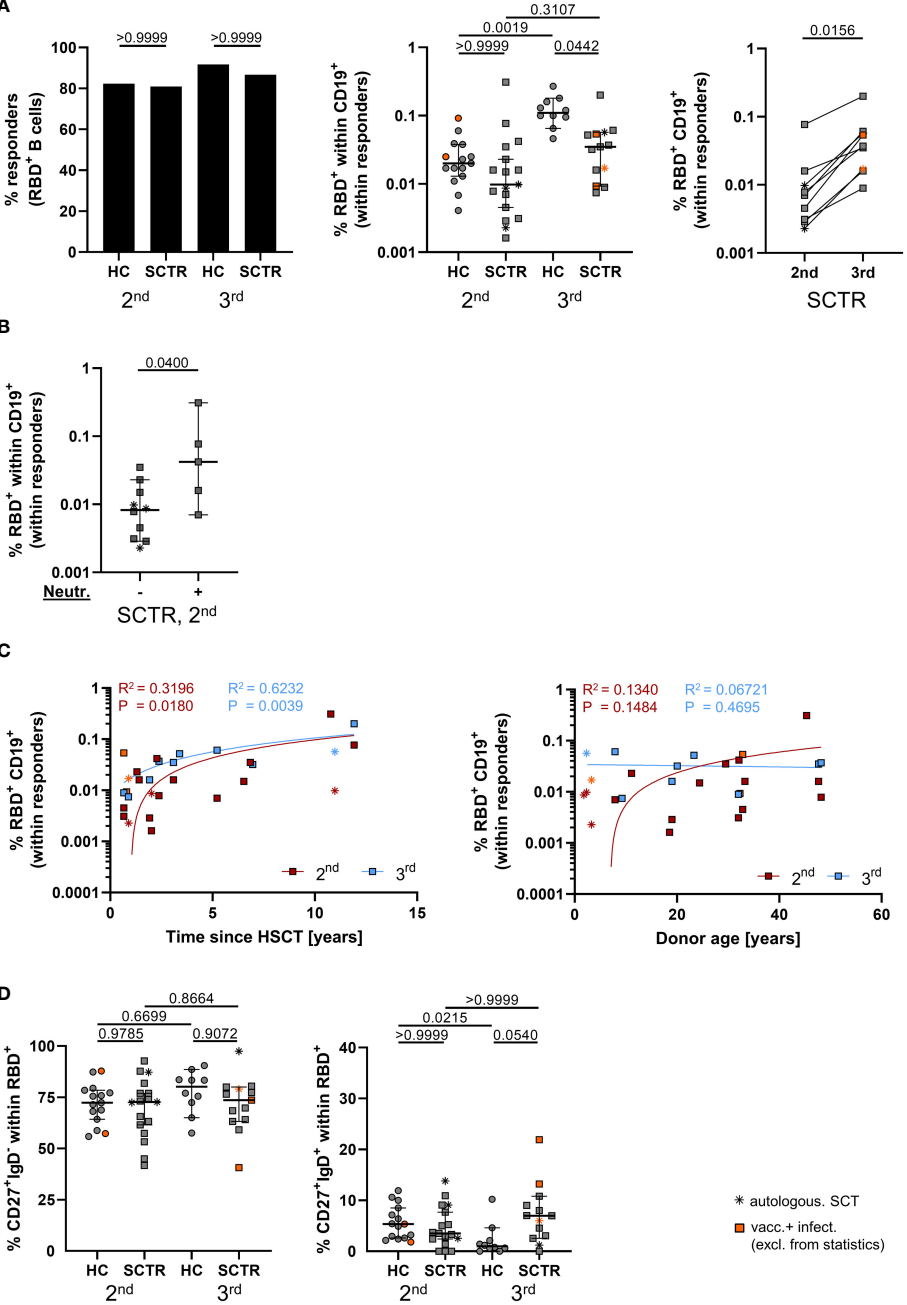
Vaccine-specific B cell immunity. **(A)** Responder rates of individuals with RBD-specific B cell responses (left), quantification of specific B cell frequencies (middle) and pairwise comparison where paired samples were available (right), as assessed by FACS. **(B)** Frequencies of specific B cells in SCTR, stratified for omicron neutralizing antibody levels above threshold or not, after the second dose. **(C)** Correlation analyses between frequencies of spike-RBD-specific B cells and time since vaccination (left) or stem cell donor age (right) in SCTR. **(D)** Quantification of isotype class-switched CD27^+^IgD^-^ (left) and CD27^+^IgD^+^ non-class-switched (right), Spike-RBD-specific memory B-cells. Graphs show medians ± 95% CI. Patients after autologous SCT are marked as indicated. Vaccinated plus infected individuals are marked in orange; they were excluded from statistical analysis.

Overall, absolute counts of CD19^+^ B cells were within the normal range for the majority of patients (**Supplemental Table 2**). Spike RBD-specific B cells were detected with fluorescently labeled probes as exemplarily illustrated in **Supplemental Figure 2A** and reported earlier (16). Rates of responders showing Spike RBD-specific B cell were similar in controls and patients at both time points (**Figure 2A**, left). Frequencies substantially rose by booster vaccination in controls, but not in patients, remaining significantly diminished even after the third dose after multiple comparisons testing (**Figure 2A**, middle). In a pairwise comparison with a limited set of patient samples, RBD^+^ B cell frequencies showed a significant increase between both time points (**Figure 2A**, right). The few SCTR with neutralizing capacity above threshold after the second dose showed significantly elevated frequencies of specific B cells (**Figure 2B**). Of note, specific B cell frequencies at both time points were positively correlated with increasing time since transplantation (**Figure 2C**, left), but not with stem cell donor age (**Figure 2C**, right), specific IgG (**Supplemental Figure 3A**), IgA (**Supplemental Figure 3B**) or neutralizing capacity, with the exception of SCTR after the 2^nd^ dose (**Supplemental Figure 3C**). We did not identify differences in specific class-switched CD27^+^IgD^-^ memory B cell frequencies between groups or time points (**Figure 2D**, left). However, patients showed a clear trend towards increased portions of non-class-switched CD27^+^IgD^+^ memory B cells after booster vaccination as compared to controls (**Figure 2D**, right). Neither frequencies of class- (**Supplemental Figure 3D**) nor of non-class-switched memory B cells (**Supplemental Figure 3E**) correlated with specific IgG levels with the exception of the CD27^+^IgD^+^ subset being negatively associated with IgG after the 2^nd^ dose in SCTR.

In summary, pediatric SCTR require a third vaccine dose for development of comparable humoral responses as controls. In addition, they show quantitative and qualitative impairments within the vaccine-specific B cell compartment.

### Quantitative and qualitative assessment of vaccine-specific T cell immunity

**Figure 3:**
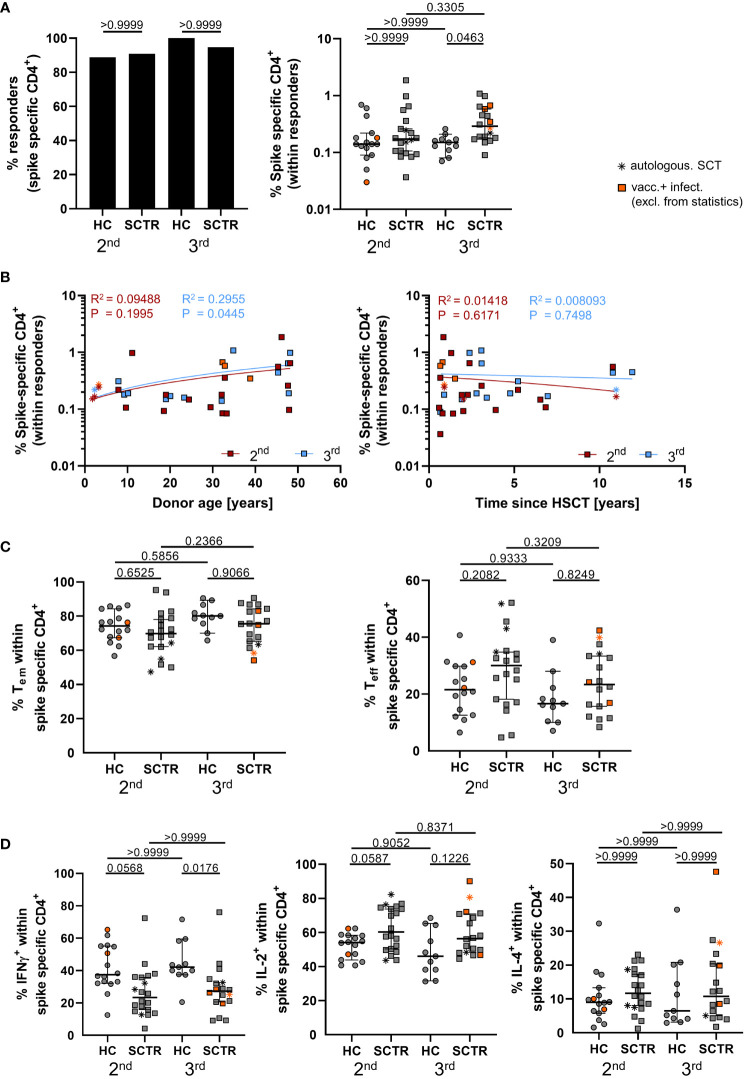
Characterization of vaccine-specific T cell immunity. PBMC from controls and SCTR were stimulated or not with Spike peptide mix. Antigen-reactive CD4^+^ T cells were detected by FACS based on CD137 and CD154 co-expression. **(A)** Illustrates responder rates (left) and frequencies (right) of Spike-specific CD4^+^ T cells. Values are background (=unstimulated control) substracted. **(B)** Correlation analysis between frequencies of vaccine-specific CD4^+^ T cells in SCTR and stem cell donor age (left) or time since transplantation (right). **(C)** Frequencies of CD45RO^+^CD62L^-^ effector/memory (left) and CD45RO^-^CD62L^-^ effector-type (right) T cells within the antigen-specific CD4^+^ compartment. **(D)** Quantification of Spike-specific CD4^+^IFNγ^+^ (left), IL-2^+^ (middle) or IL-4^+^ (right) T cells. Graphs show medians ± 95% CI. Patients after autologous SCT are marked as indicated. Vaccinated plus infected individuals are marked in orange; they were excluded from statistical analysis.

Spike-specific CD4^+^ T cells were identified as depicted in **Supplemental Figure 4A** according to co-expression of CD137 and CD154 after peptide mix stimulation, as reported earlier (17–19). Numbers of individuals showing CD4^+^ T cell responses were not substantially different between groups (**Figure 3A**, left). Interestingly, SCTR showed significantly higher frequencies of specific T cells than healthy individuals after booster vaccination (**Figure 3A**, right). The magnitude of responses in patients significantly correlated with stem cell transplant donor age after the third dose (**Figure 3B**, left), but not with time since transplantation (**Figure 3B**, right). Frequencies of specific CD4^+^ T cells did not correlate with IgG levels for any of the groups (**Supplemental Figure 5A**), but with IgA for HC after the second dose (**Supplemental Figure 5B**) and neutralizing capacity levels in SCTR after the second dose (**Supplemental Figure 5C**). Both groups did not exhibit differences with respect to frequencies of specific CD45RO^+^CD62L^-^ effector/memory- or CD45RO^-^CD62L^-^ effector-type T cells (**Figure 3C**). However, functional analysis revealed diminished portions of CD4^+^IFNγ^+^ T cells after the second and third dose in patients compared to controls (**Figure 3D**, left). We further noted the opposite trend for IL-2, as reflected by increased frequencies in SCTR at both time points (**Figure 3D**, middle). Neither of these observations did apply to frequencies of Spike-specific IL-4^+^ T cells (**Figure 3D**, right). Polyfunctionality analysis did not reveal appreciable differences in frequencies of cells secreting none, one, two (**Supplemental Figure 5D**, left), all three (**Supplemental Figure 5D**, left and right) or any of the three cytokines (**Supplemental Figure 5E**) between groups.

We only noted low responder rates with respect to Spike-specific CD8^+^ T cells (**Supplemental Figure 5F**, left) that were detected based on CD137 and IFNγ co-expression (**Supplemental Figure 4**), as shown before (20). Therefore, we did not conduct statistical analysis for comparing frequencies in patients and controls (**Supplemental Figure 5E**, right). Absolute CD8 counts were consistently within the normal range (**Supplemental Table 2**), therefore not providing an obvious explanation for the low responder rates observed.

In summary, SCTR are characterized by elevated frequencies of vaccine-specific CD4^+^ T cells showing a functional programming towards increased IL-2- at the expense of anti-viral IFNγ production.

### Vaccine-specific immune features in SCTR experiencing GvHD

Given the small size of our pediatric cohort, further stratification of patients for assessment of distinct immunological features was only meaningful for patients experiencing GvHD or not, representing the largest subgroups, respectively. It is noteworthy that as opposed to all previous analyses, also patients with breakthrough infections during the study were included in statistical evaluations. Importantly, SCTR with GvHD developed significantly lower IgA and neutralizing antibody levels after the second dose, an effect that disappeared after the third dose (**Supplemental Figure 6A**). We did not observe significant differences with respect to vaccine-specific CD4^+^ and B cell frequencies between groups (**Supplemental Figures 6B, C**).

## Discussion

Whereas multiple studies have addressed standard SARS-CoV2 mRNA-vaccination induced immunity in adult hematopoietic stem cell transplant recipients, information on pediatric patients, particularly after booster vaccination, is scarce. Our data demonstrate that young stem cell transplant recipients, similar to their adult counterparts (7), substantially benefit from a third dose with respect to all humoral parameters analyzed. This particularly applies to the commonly disregarded assessment of neutralization capacity, with Omicron representing the predominant variant within the time frame of our study and being considered an enduring threat for stem cell transplanted individuals (21). Our findings therefore underpin current recommendations of expert consortiums, proposing early boosters or 3-dose primary vaccination courses for this at-risk group (22). In the context of SARS-CoV2 wildtype infection, IgA production has been found to significantly contribute to virus neutralization particularly in the early disease phase (23). An association between wildtype virus neutralizing capacity and anti-wildtype IgA levels was also noted after SARS-CoV2 vaccination (24). The fact that we did not identify significant correlations between IgA and neutralization capacity might depend on the different wildtype vs. Omicron variants included in the respective ELISA kits (25), therefore not allowing us to determine the individual IgA contribution to omicron neutralization. It is nevertheless interesting that 2 of the 3 individuals with low neutralizing antibody levels were also negative for IgA in our study. Of note, we observed comparably homogenous humoral response outcomes despite the heterogeneity of primary diseases and small size of our patient group, being characteristic for pediatric vaccination studies (16, 26). Meta-analyses of data from multiple adult SCTR cohorts demonstrated that SARS-CoV2 vaccination-induced antibody levels are primarily impacted by time since transplantation, GvHD status and immunosuppressive medication (3, 27), with the last aspect not applying to the majority of our patients. On the background of vaccination recommendations, it is noteworthy that humoral titers normalized from second to third vaccine dose also in our pediatric GvHD-subgroup, again supporting the importance of booster vaccinations. Although an association between neutralizing antibody levels and time since transplantation was not evident in our study, increase of the latter was significantly associated with rising frequencies of Spike-specific B cells. This observation might be explained by the comparably slow post-transplant reconstitution kinetics of the B cell compartment (28). Overall, vaccine-specific B cells remained quantitatively impaired particularly after the third dose and showed an expansion of the non-isotype-switched CD27^+^IgD^+^ memory subset, both features described earlier for adult and pediatric kidney transplant recipients under immunosuppressive medication (16, 18) and reported for bulk B cells after SCT (29). It needs to be determined whether such maturation defects remain long-term and also include differentiation into long-lived, vaccine-specific plasma cells residing in the bone marrow (30), providing sustained antibody production in healthy individuals (31). In line with previous studies on both healthy adults and solid organ transplant recipients (20) as well as adult SCTR (32), CD8 responder rates after vaccination were comparably low with no significant differences between controls and SCT patients. We can only speculate that this might in part be related to a slightly lower efficacy of the employed overlapping 15-mer peptides to activate CD8^+^ T cells via MHC-I molecules (33).

Of note, we observed similar frequencies of Spike-specific CD40L^+^CD137^+^ CD4^+^ T cells in patients and controls after the second dose. Using a similar approach, Federico et al. (32) detected diminished portions of specific cells in adult SCTR. Paediatric patients with preconditions generally mount more robust T cell responses (16) than their adult counterparts (20) and patient age has been generally determined to critically impact SARS-CoV2 vaccination outcomes in SCTR (5). Furthermore, the difference in cellular responses reported in our study and others (32) might also be explained by patient cohort composition, considering acute or chronic GvHD in about 50% of their patients. Surprisingly, we detected significantly elevated frequencies of specific CD4^+^ T cells in patients vs. controls after the booster dose. This increase was accompanied by a trend towards higher portions of IL-2- and significantly diminished frequencies of IFNγ- secreting cells at both time points with the latter aspect being supported by data from adult SCTR cohorts (10). Importantly, all aforementioned features have been reported earlier for SARS-CoV2-specific T helper cells isolated from COVID-19-patients with certain pre-conditions, including co-morbidities or advanced age (17). Other studies have complemented these findings by showing that individuals suffering from severe disease characteristically harbor SARS-CoV2-specific T cells with reduced functional avidity (34). It is therefore tempting to speculate that the reduced anti-viral capacity of vaccine-induced, low-avidity T cells, reflected by impaired IFNγ secretion, is compensated by augmented IL-2 driven cellular expansion in at risk-groups by yet unidentified driving forces. Although we noted a correlation between progressing stem cell donor age and increasing frequencies of vaccine-specific T cells, it appears more likely that it is rather the sum of pre-conditions of the stem cell recipient shaping the cells´ attenuated functional potential.

Limitations of our study include the comparably small cohort size without the possibility for further subgroup stratification and long-term follow-up that is explained by ethical guidelines restricting sampling of high blood quantities required for cellular analyses from pediatric patients. Furthermore, there was only a small window of opportunity between availability of mRNA vaccines for children younger than 12 or 5 years, respectively, and high dissemination of the omicron variant, reflected by some breakthrough infections in our cohort, thereby complicating recruitment of more virus-naïve individuals.

Nevertheless, our data not only identify multiple impairments of cellular immunity in pediatric SCTR; more importantly, they highlight the requirement of a third dose for mounting sufficient humoral responses, including Omicron neutralization. On the background of vaccination hesitancy amongst parents of SCTR (35), transplant pediatricians are urged to provide COVID-19-related education and information to protect this vulnerable patient group.

## Materials and methods

### Study design and medication

This observational study was conducted between January and June 2022 with pediatric stem cell transplant recipients (SCTR) recruited from the Charité Department of Pediatric Oncology and Hematology. Age-matched healthy controls were largely siblings of patients and not diagnosed with any acute or chronic medical condition. Basic characteristics of both groups are depicted in **Table 1**, whereas further details of enrolled patients are included in **Supplemental Table 1**. SARS-CoV-2 specific humoral and/or cellular responses were analyzed after second and third vaccination. Vaccination data, including vaccination type, number of doses, and vaccination dates were obtained from participants at visits. All individuals were vaccinated with the SARS-CoV2 vaccine BNT162b2 (Comirnaty; BioNTech/Pfizer) according to the manufacturer´s guidelines: all persons aged 12 and above were vaccinated with 30 µg. For children aged 5-11, the dosage was adjusted to 10 µg. Infants and children aged from 6 months to 4 years were vaccinated with the recommended dose of 3 µg.

**Table 1:** Basic characteristics of SCT patients and healthy controls.

Variable	SCTR	HC	*P value*
n	29	29	
Median age at vaccination, years (range)	9.8 (2.1-19.8)	12.0 (3.0-19.0)	0.2909
Females, n (%)	17 (58.6)	12 (41.4)	0.2935
Median time from 2^nd^ vaccination to sampling, days (range)	35.0 (20-62)	38.0 (20-54)	0.8665
Median time from 3^rd^ vaccination to sampling, days (range)	33.0 (21-58)	33.0 (18-57)	0.6810
Median age at SCT, years (range)	5.4 (0.2-16.4)	n/a	
Median time since SCT, years (range)	2.3 (0.6-11.9)	n/a	
Indication for SCT, n (%) Lymphoproliferative malignancies Myeloid disease Solid tumors Immunodeficiency syndrome	14 (48.3) 7 (24.1) 5 (17.2) 3 (10.3)	n/a	
Donor type, n (%) MUD MMRD MSD Autologous	14 (48.3) 7 (24.1) 5 (17.2) 3 (10.3)	n/a	
Stem cell source, n (%) PBSC BM CB	18 (62.1) 10 (34.5) 1 (3.4)	n/a	

SCTR, stem cell transplant; HC, healthy control; MMRD, mismatched related donor; MUD, matched unrelated donor; MSD, matched sibling donor; n/a, not applicable.

Blood and serum samples were collected approximately 5 weeks after vaccinations with no significant differences between groups. The local ethics committee of the Charité Universitätsmedizin Berlin, Germany (EA2/227/21) approved this study, and all volunteers and/or their legal guardians provided informed consent to participate.

### Analysis of humoral immunity

Previous SARS-CoV2 infection during the study was assessed based on medical history, frequent point of care antigen testing and a SARS-CoV2 nucleoprotein specific ELISA (Euroimmun). Levels of SARS-CoV2 S1 domain specific IgG (QuantiVac, Euroimmun) and IgA (Euroimmun) were also determined by ELISA. Results for IgA and nucleoprotein were calculated as the O.D. ratio of the respective sample over the O.D. of the calibrator provided with the ELISA kit. An O.D. ratio of >1.1 was considered positive, of >0.8 to <1.1 borderline, and of <0.8 as negative. Serum samples exceeding O.D. ratios of 8 were re-measured after pre-dilution. Results for Spike S1 domain specific IgG were provided as BAU/ml and considered positive when above the threshold of ≥35.2 BAU/ml. To quantify virus neutralizing antibodies against SARS-CoV2 Omicron variant, a competitive binding ELISA (sVNT kit, GenScript) was used. In this assay, neutralizing serum antibodies compete for binding to recombinant, horseradish peroxidase-labeled SARS-CoV2 RBD (Omicron variant) with human angiotensin-converting enzyme 2 receptor protein. According to the manufacturer´s instructions, a neutralizing capacity above 30% was considered positive.

### Characterization of SARS-CoV2 vaccine-specific B and T cells

PBMCs were isolated from EDTA blood using Lympho24+ Spin Medium (pluriSelect, Leipzig) by means of density gradient centrifugation. For analysis of SARS-CoV2 vaccine-specific B cells by flow cytometry, 5×10^6^-1×10^7^ mononuclear cells were stained with the antibodies listed in **Supplemental Table 3**. Among B cells, gated as single live CD19^+^CD3^-^CD14^-^CD56^-^ lymphocytes (**Supplementary Figure 2A**), vaccine-specific B cells were detected by co-staining with fluorescently labeled recombinant RBD protein and recombinant full spike protein (both alpha-variant, RnD Systems, Minneapolis, MN, United States) as described previously (18, 19). This double labeling strategy was chosen also in agreement with other groups to reduce background staining (36, 37).

Vaccine-specific T cells were identified after stimulation of 3 × 10^6^ PBMCs for 16h at 37°C and 5% CO_2_ with overlapping 15-mer peptides covering the complete SARS-CoV2 spike protein (alpha-variant, JPT, Berlin, Germany) at a final concentration of 0.5 µg/ml per peptide as described earlier(17). Equal quantities of DMSO as contained in the peptide mix were added to unstimulated controls. For intracellular molecule retention, Brefeldin A was added at a final concentration of 10 µg/ml. After stimulation, cells were stained with a surface antibody mixture at room temperature for 20 minutes, followed by fixation and permeabilization (FACS Lysing Solution/FACS Perm II Solution, BD Bioscience, Heidelberg, Germany) and staining with antibodies against intracellular molecules (**Supplemental Table 4**). Identification of antigen-reactive CD4^+^ T cells was based on CD137 and CD154 co-expression (**Supplementary Figure 4**). When stimulated samples contained a minimum of twenty events and at least twofold higher frequencies of CD137^+^CD154^+^ T cells compared to the respective unstimulated control (stimulation index of 2), a response was considered positive. Cells were measured with a BD FortessaX20 flow cytometer (BD Bioscience, Heidelberg, Germany).

### Data analysis and statistics

FlowJo 10 (BD) was used for analysis of flow cytometric data. Frequencies of spike-specific CD4^+^ T cells were background (unstimulated control) subtracted. Boolean gating in FlowJo was used for quantification of cytokine co-expression. Statistical analysis and graph preparation was performed in GraphPad Prism 8 (Graphpad, San Diego, CA, USA). Data was tested for normal distribution with the Kolmogorov-Smirnov test. In case of normal distribution an ordinary one-way ANOVA with consecutive Sidak’s multiple comparison test was used. When data sets did not show normal distribution, a Kruskal-Wallis with Dunn’s multiple comparison was applied. For comparison of two groups a Mann-Whitney test was performed. Analysis of contingency tables was conducted by applying Fisher´s exact test (**Supplemental Table 5**). Statistical significance was considered for *p*>0.05.

## Data availability statement

The original contributions presented in the study are included in the article/**Supplementary Material**. Further inquiries can be directed to the corresponding authors.

## Ethics statement

The studies involving humans were approved by Charité Universitätsmedizin Berlin Ethics Commission (EA2/227/21). The studies were conducted in accordance with the local legislation and institutional requirements. Written informed consent for participation in this study was provided by the participants’ legal guardians/next of kin.

## Author contributions

LMT Conceptualization, Investigation, Formal analysis, Visualization, Writing. AS, Conceptualization, Methodology, Supervision, Visualization, Writing, Review. KK, Conceptualization, Funding acquisition, Supervision, Writing, Review. JS, Conceptualization, Resources. AE, Resources. LT, VP, CS, JS, AK, AW, ES and CL, Methodology, Investigation, Review. CE, Investigation. GH, Project administration, HS, Resources, Investigation and Review. All authors contributed to the article and approved the submitted version.
