# Supplementary material for: Impact of a booster dose on SARS-CoV2 mRNA vaccine-specific humoral-, B- and T cell immunity in pediatric stem cell transplant recipients
Source: Front Immunol. 2023 Oct 24;14:1239519. doi: 10.3389/fimmu.2023.1239519 (PMC10628529; doi:10.3389/fimmu.2023.1239519)
Supplement: Supplementary file 1 [file DataSheet_1.pdf]

Thole et al. Supplemental Material

Table of Content

**Supplemental Figure 1**	Correlation of neutralizing capacity with donor age, time since transplantation and specific IgA/IgG levels
**Supplemental Figure 2**	Identification and phenotypic characterization of SARS-CoV-2 specific B cells
**Supplemental Figure 3**	Specific B cell correlation analyses
**Supplemental Figure 4**	Detection and characterization of SARS-CoV2 Spike-specific T cells
**Supplemental Figure 5**	$CD4^+$ T cell correlation analyses, polyfunctionality and CD8 responses
**Supplemental Figure 6**	Vaccine-specific immunity in patients stratified for GvHD status
**Supplemental Table 1**	Detailed characteristics of SCTR
**Supplemental Table 2**	Absolute T- and B cell counts for SCTR
**Supplemental Table 3**	Antibodies for phenotypic analysis of B cells
**Supplemental Table 4**	Antibodies for functional analysis of T cells
**Supplemental Table 5**	Statistics

Thole et al. Supplemental Figure 1

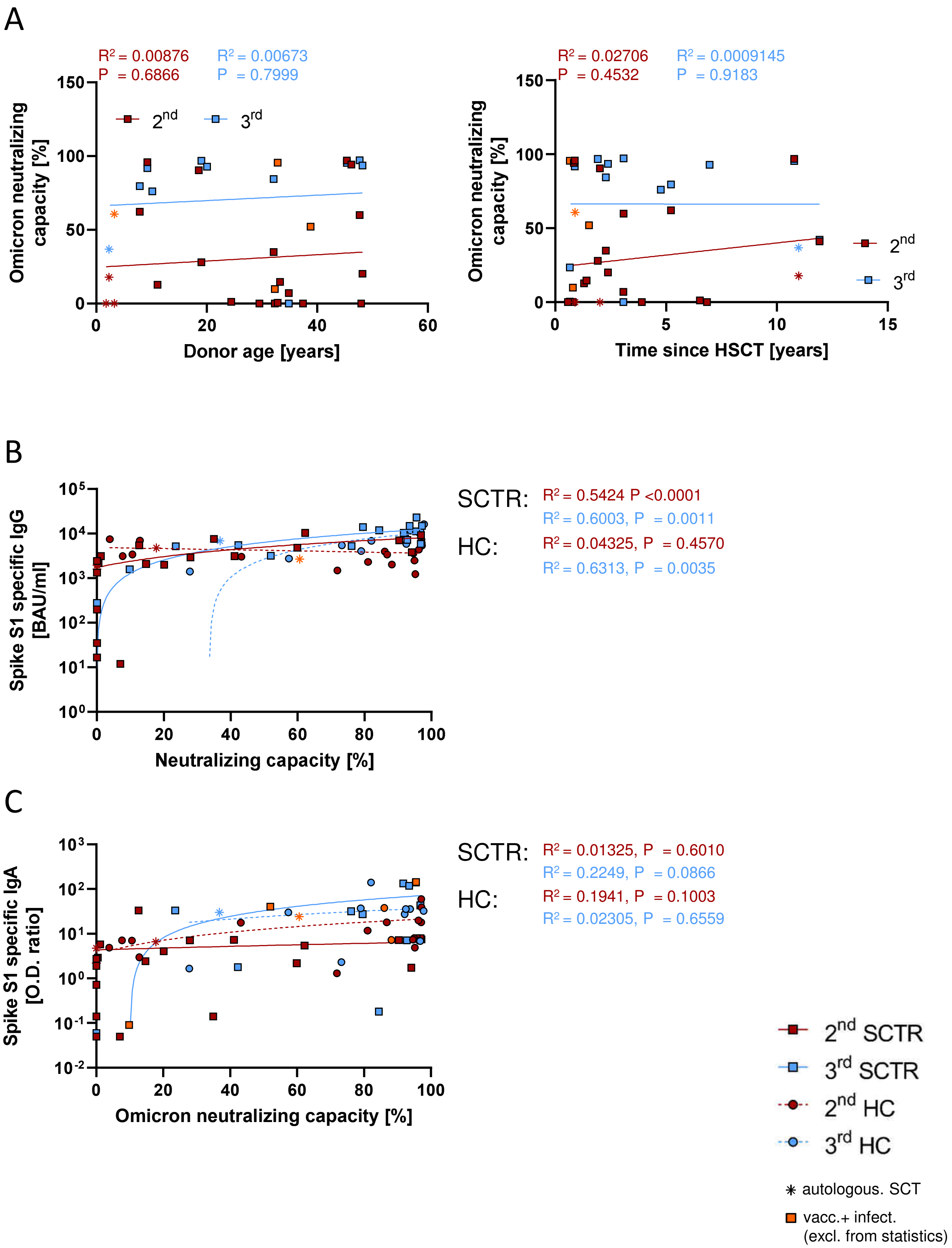

Correlation of neutralizing capacity with donor age, time since transplantation and specific IgA/IgG levels. Neutralizing capacity against Omicron variant as determined by ELISA was correlated with stem cell donor age (A, left), time since transplantation (A, right) in SCTR and with specific IgG- (B) or IgA (C) levels in patients and controls. Patients after autologous SCT are marked as indicated. Vaccinated plus infected individuals are marked in orange; they were excluded from statistical analysis.

Thole et al. Supplemental Figure 2

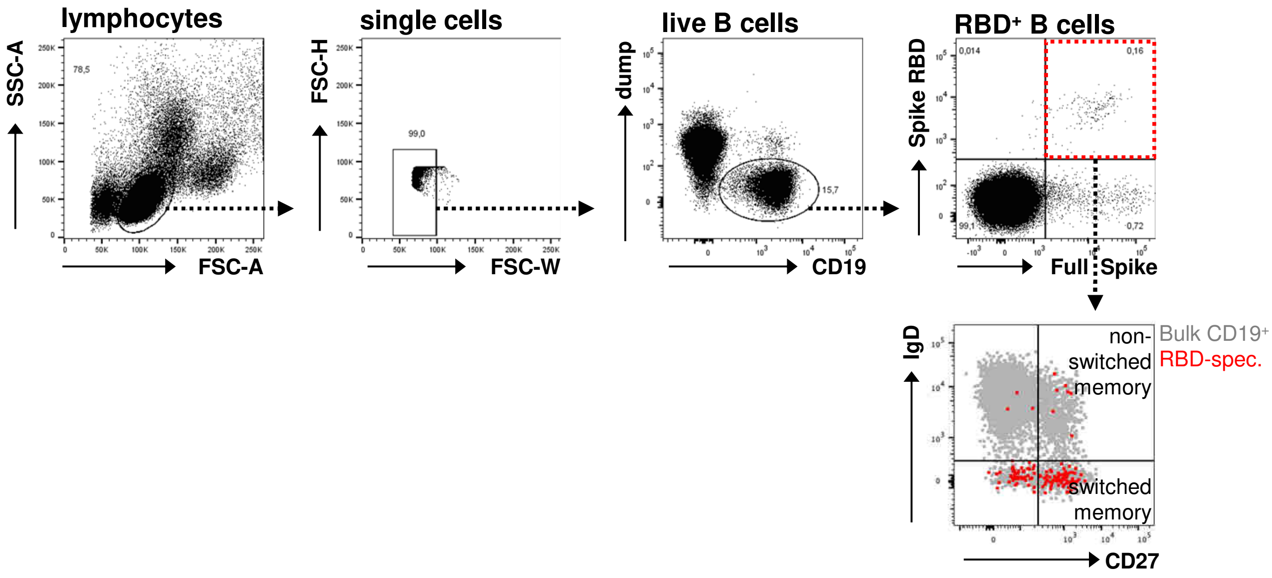

Identification and phenotypic characterization of SARS-CoV-2 specific B cells. Antigen-reactive live single CD14$^-$CD56$^-$CD3$^-$ ("dump" negative) CD19$^+$ B cells were detected by FACS according to co-staining with recombinant Spike RBD-FITC and recombinant full spike-APC. Specific cells were further analyzed for memory differentiation based on CD27 and IgD expression with subsets defined as indicated.

Thole et al. Supplemental Figure 3

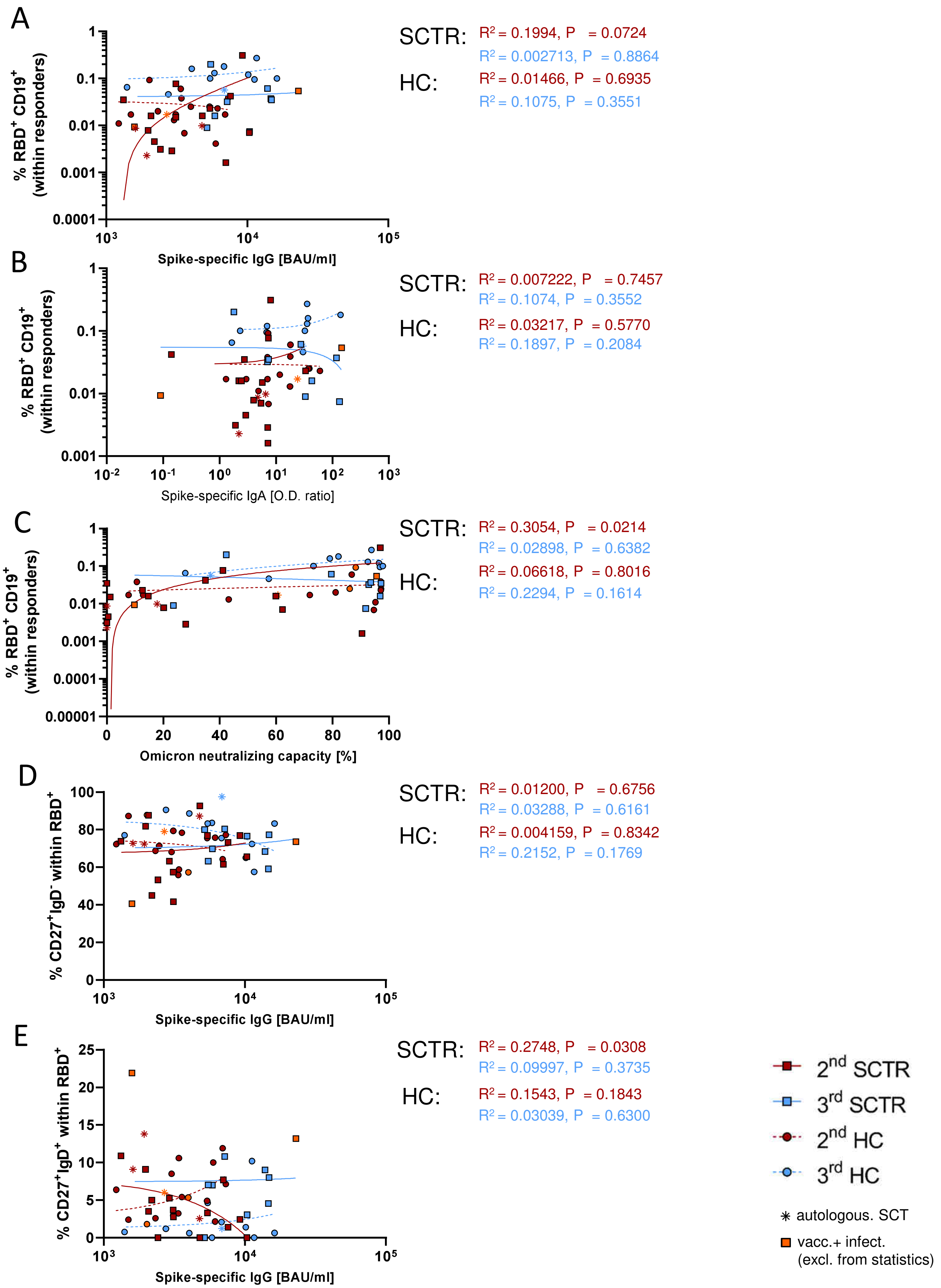

Specific B cell correlation analyses. Specific B cells were identified as before; frequencies were correlated in SCTR and HCs with specific IgG**-** (A), IgA- (B) or neutralizing antibody levels (C). Correlation analysis of specific $CD27^+IgD^-$ isotype class switched (D) or $CD27^+IgD^+$ non-switched B cells (E) with specific IgG levels. Patients after autologous SCT are marked as indicated. Vaccinated plus infected individuals are marked in orange; they were excluded from statistical analysis.

Thole et al. Supplemental Figure 4

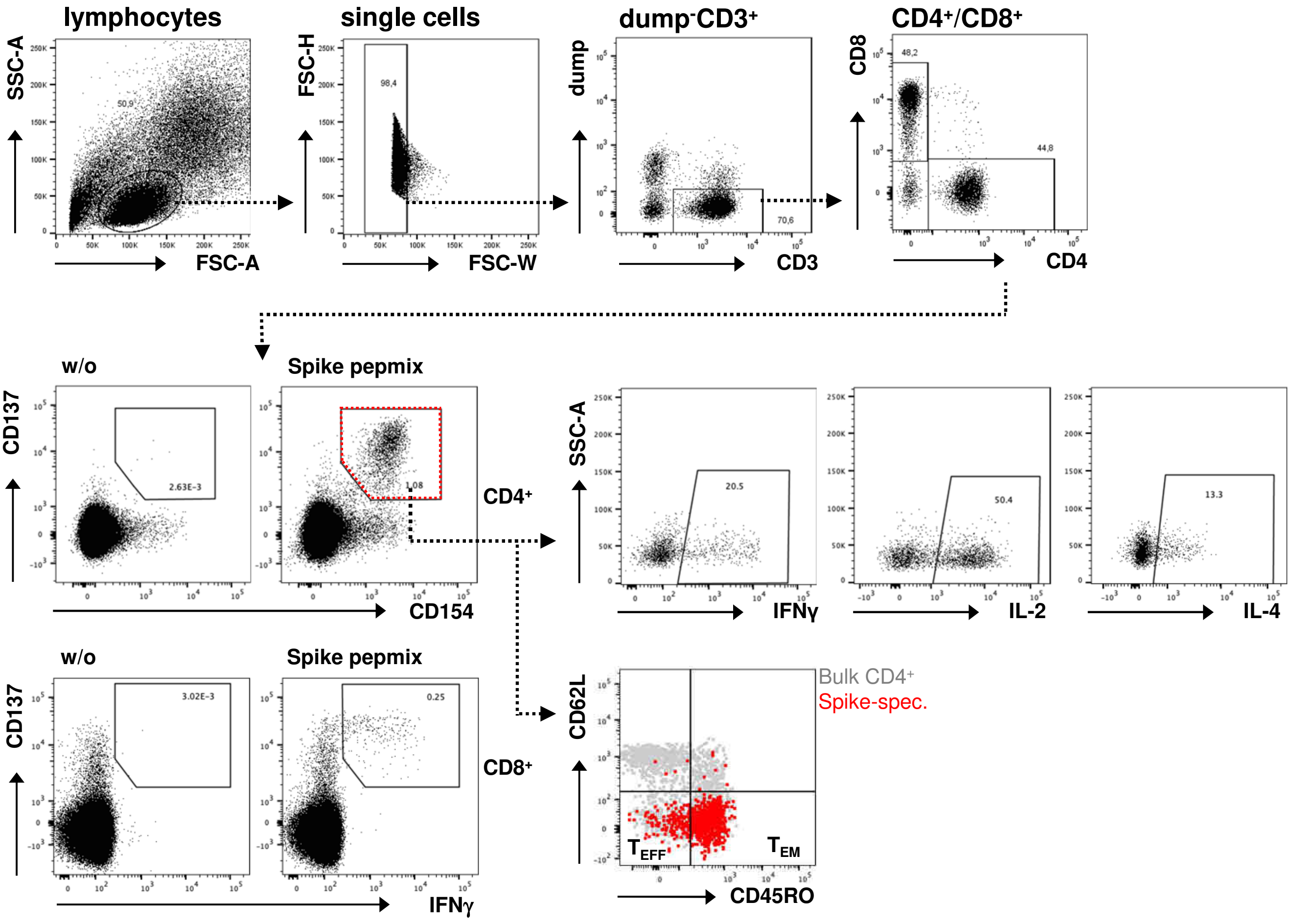

Detection and characterization of SARS-CoV2 Spike-specific T cells. PBMC were stimulated for 16h with SARS-CoV2 Spike peptide mix or left untreated. Antigen-specific live single $CD14^{-}CD19^{-}$("dump" negative) $CD3^{+}CD4^{+}$ T cells were identified by FACS according to co-expression of CD137 and CD154. Cells were further analyzed for memory differentiation ($CD45RO^{+}CD62L^{-}$ - effector/memory (T_{EM}); $CD45RO^{-}CD62L^{-}$ - effector-type (T_{EFF}) or cytokine production as depicted.

Thole et al. Supplemental Figure 5

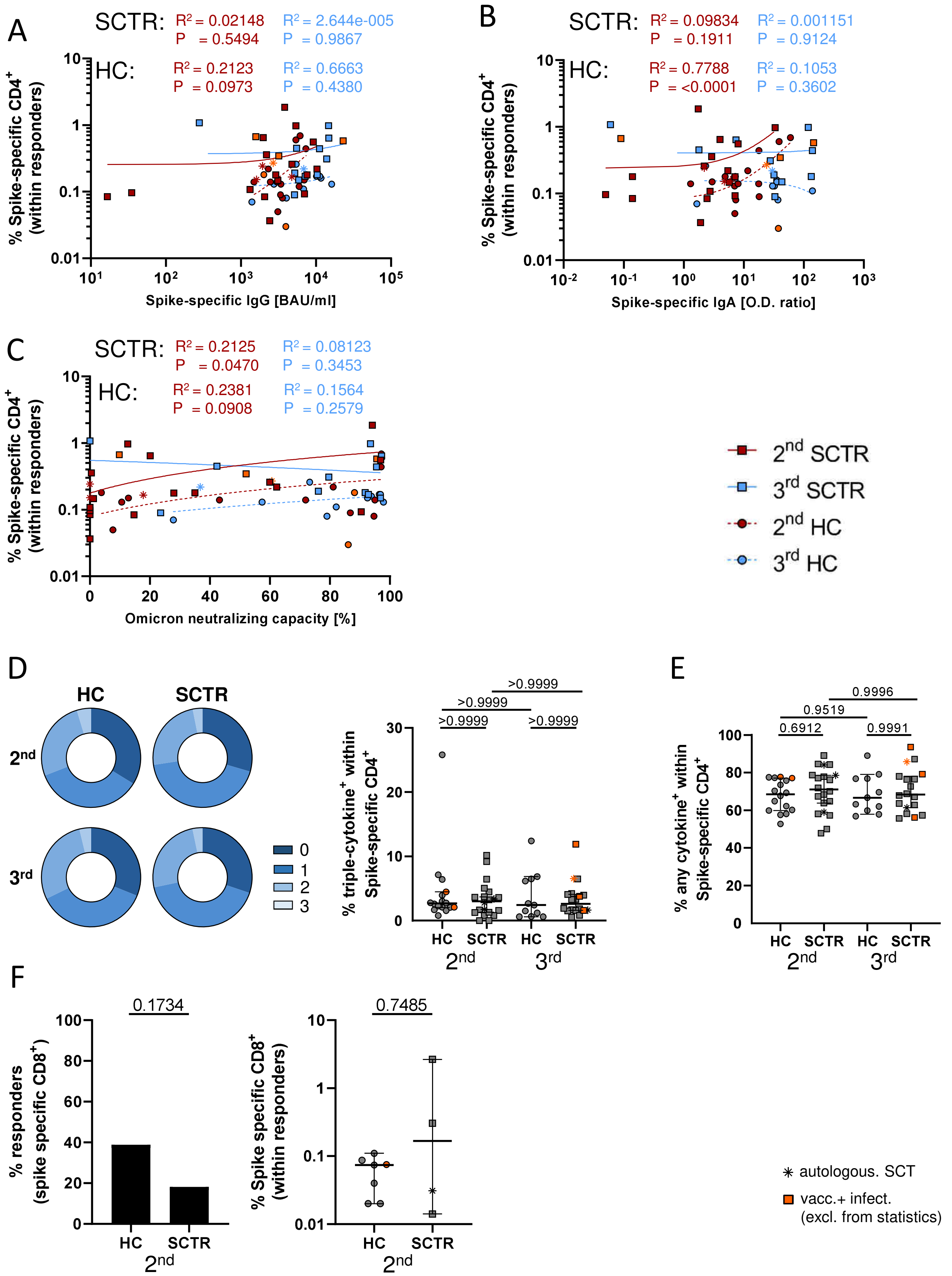

CD4⁺ T cell correlation analyses, polyfunctionality and CD8 responses. Frequencies of vaccine-specific CD4⁺ T cells were determined as before and correlated with specific IgG- (A), IgA- (B) or neutralizing antibody levels (C). (D) Polyfunctional analysis of specific CD4⁺ T cells secreting 0, 1, 2 or 3 of the cytokines IFNγ, IL-2 and/or IL-4 based on the respective means (left) and statistical analysis thereof (right). (E) Frequencies of specific CD4⁺ T cells secreting any of the three cytokines. (F) Spike-specific CD3⁺CD8⁺ T cells were detected based on CD137 and IFNγ co-expression. Depicted are percentages of individuals with a CD8⁺ T cell response (left) and frequencies of specific CD8⁺ T cells (after background substraction) within responders (right) after the second vaccine dose. Patients after autologous SCT are marked as indicated. Vaccinated plus infected individuals are marked in orange; they were excluded from statistical analysis.

Thole et al. Supplemental Figure 6

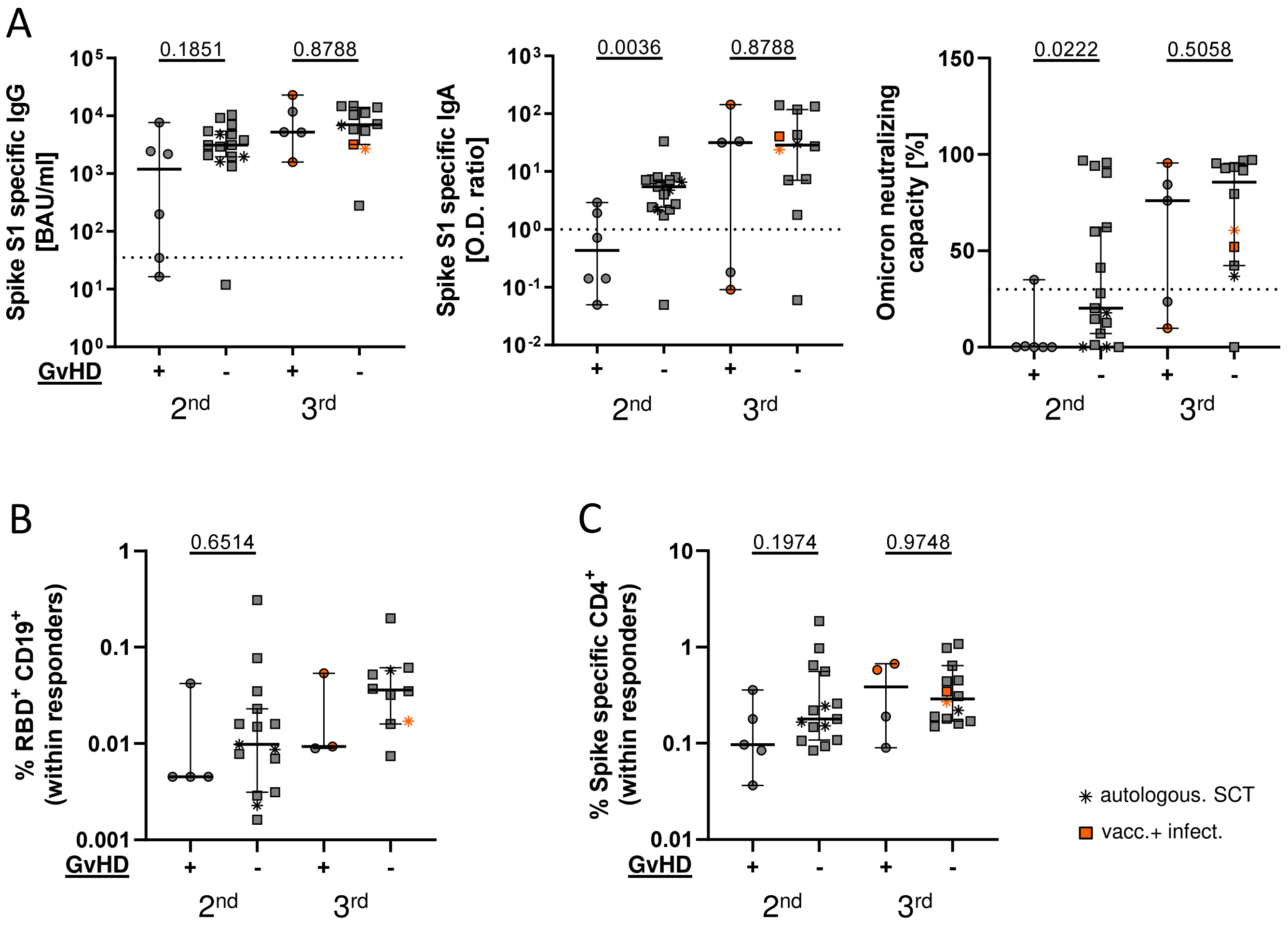

Vaccine-specific immunity in patients stratified for GvHD status. SCTR were stratified according to experiencing GvHD or not. Specific IgG (A, left), IgA (A, middle), neutralizing capacity levels (A, right), frequencies of specific B cells (B) or T cells (C) were determined in both subgroups after second or third vaccine dose, respectively.

Supplemental Table 1: Detailed characteristics of SCTR

ID	gender	Underlying disease	Age (years)	Time since SCT (years)	Previous SCT	Donor type	Donor age (years)	Stem cell source	Conditioning	GvHD[$]	Ongoing treatment[§]
**1**	F	SAA	15,8	10,8	no	MMRD	45,4	PBSC	Flu/TT/ATG	no	no
**2**	F	NB	6,2	1,5	yes (2020/10, 2019/8)	MMRD	38,8	PBSC	Flu/TT/Mel/ATG	no	no
**3**	M	B-NHL	9,1	2,3	no	MUD	32,1	PBSC	Flu/Tax/Carb/ Mtx/ATG	pulmonary GvHD °I	inhaled CS
**4**	M	B-ALL;	12,3	6,8	no	MUD	29,5	PBSC	TBI(+skull)/ Eto/ATG	no	no
**5**	M	NB	13,3	11,0	no	Auto	2,3	PBSC	Mel/Eto/Carb	no	no
**6**	M	MDS	18,1	2,8	no	MUD	47,9	PBSC	Flu/Treo/ATG	no	no
**7**	F	B-ALL	5,7	0,6	no	MUD	37,4	BM	TBI/Eto/ATG	intestinal GvHD °II-III; GvHD skin °I	Eve/MPA; ECP; 2×/mo Vedolizumab 2×300 mg/mo i.v.
**8**	M	B-ALL	10,1	3,1	yes (2019/1, 2018/1)	MMRD	34,9	PBSC	Flu/TT/Mel/ATG	no	Dasatinib 20 mg/d
**9**	F	B-ALL	12,6	6,5	no	MUD	24,4	BM	Bu/Eto/Cy/ATG	no	no
**10**	M	SCID	2,2	2,0	no	MUD	18,5	PBSC	Flu/Threo/ATG	no	no
**11**	M	B-ALL	7,2	0,9	no	MSD	9,2	PBSC	TBI/Eto	no	no
**12**	F	B-ALL	8,7	0,8	no	MUD	32,3	BM	TBI/Eto/ATG	GvHD skin °I	no
**13**	M	B-ALL	19,8	3,4	no	MUD	23,3	PBSC	Treo/Cyc/TT/ATG	no	no
**14**	M	NB	3,8	2,0	no	Auto	1,8	PBSC	Bu/Mel	no	no
**15**	F	AML	2,1	1,4	no	MMRD	33,3	PBSC	Flu/TT/Treo/ATG	no	no
**16**	M	B-ALL	7,3	1,9	no	MUD	19,0	PBSC	Eto/ATG	no	no
**17**	M	T-ALL	16,8	1,3	no	MSD	11,1	BM	TBI/Eto	no	no
**18**	F	NB	6,9	2,4	no	MMRD	48,2	PBSC	Bu/Mel/Flu/ATG	no	Alectinib 450 mg/d
**19**	F	B-ALL	12,7	0,7	no	MUD	32,9	BM	TBI/Eto/ATG	GvHD skin °I	no
**20**	M	T-ALL	11,3	0,8	yes (2021/3, 2018/12, 2017/9)	MMRD	46,2	PBSC	Flu/Treo/TT/ATG	no	DLI; Venetoclax
**21**	F	MDS	18,3	4,8	no	MSD	10,2	BM	Flu/TT/ATG	GvHD skin °I	no
**22**	M	AML;	16,5	7,0	no	MUD	20,1	BM	TBI/AraC/GO/Flu/ Amsa/Cyc/ATG	no	no
**23**	F	MDS	12,0	5,2	no	MSD	7,9	BM	Flu/TT/Mtx	no	no
**24**	F	B-ALL	5,3	4,2	yes (2018/3, 2017/11)	MUD	47,7	PBSC	Flu/TT/Treo/ ATG	GvHD skin °III; pulmonary GvHD °I	Cutaquig 2 g/w s.c; inhaled CS Montelukast 4 mg/d
**25**	M	bThal	2,9	0,6	no	MSD	9,7	BM	Bu/Thio/Cyc	no	no
**26**	M	B-ALL	9,8	3,1	no	MMRD	47,7	PBSC	Flu/TT/Mel/ATG	no	no
**27**	M	WAS	3,3	0,7	no	MUD	32,0	BM	Flu/Bu/ATG	GvHD skin °I	no
**28**	F	NB	4,2	0,9	no	Auto	3,3	PBSC	Bu/Mel	no	no
**29**	M	T-B+SCID (y-chain)	12,4	11,9	no	MUD	0	CB	unknown	no	no
Median (Range)			9.8 (2.1-19.8)	2.4 (0.6-11.9)			29.5 (0-48.2)				

SCT – stem cell transplantation; SAA – severe aplastic anemia; NB – neuroblastoma; NHL – non-Hodgkin lymphoma; ALL – acute lymphoblastic leukemia; MDS – myelodysplastic syndrome; SCID – severe combined immunodeficiency; AML – acute myeloblastic leukemia; bThal – β-thalassaemia; WAS – Wiskott-Aldrich syndrome; MMRD – mismatched related donor; MUD – matched unrelated donor; Auto – autologous; MSD – matched sibling donor; PBSC – peripheral blood stem cells; BM – bone marrow; CB – cord blood; Flu – fludarabine; TT – thiotepa; ATG – anti-thymocyte globulin; Mel – melphalan; Carb – carboplatin; Mtx – mitoxantrone; Eto – etoposide; Treo – treosulfan; Bu – busulfan; AraC – cytosine arabinoside; GO – gemtuzumab ozogamicin; Amsa – amsacrine; Cyc – cyclophosphamide; TBI – total body irradiation; GvHD – graft versus host disease; Eve – everolimus; MPA – mycophenolic acid; ECP – extracorporeal photopheresis; CS – corticosteroids. [$]between first vaccination and time of humoral and cellular analysis. [§]Ongoing GvHD and/or treatment of underlying disease at time of humoral and cellular analysis

Supplemental Table 2: Absolute T- and B cell counts for SCTR

ID	CD4⁺ T cells/nl			CD8⁺ T cells/nl			CD4⁺/CD8⁺ ratio			CD19⁺ B cells/nl		
	reference range	count/nl 2nd	count/nl 3rd	reference range	count/nl 2nd	count/nl 3rd	reference range	count/nl 2nd	count/nl 3rd	reference range	count/nl 2nd	count/nl 3rd
**1**	0.4-2.1	0.46	0.15	0.2-1.2	0.49	1.16	0.9-3.4	0.90	0.10	0.20-0.6	0.11	0.16
**2**	0.3-2.0	-	0.71	0.3-1.8	-	0.50	0.9-2.6	-	1.40	0.2-1.6	-	0.33
**3**	0.3-2.0	0.79	0.81	0.3-1.8	0.52	0.48	0.9-2.6	1.50	1.70	0.2-1.6	0.31	0.32
**4**	0.4-2.1	1.00	-	0.2-1.2	1.10	-	0.9-3.4	0.90	-	0.2-0.6	0.38	-
**5**	0.4-2.1	1.04	0.83	0.2-1.2	0.54	0.47	0.9-3.4	1.90	1.80	0.2-0.6	0.5	0.49
**6**	0.5-1.2	-	0.72	0.3-0.8	0.53	-	1.1-3.0	1.30	-	0.1-0.4	0.33	-
**7**	0.3-2.0	0.21	-	0.3-1.8	1.11	-	0.9-2.6	0.20	-	0.2-1.6	0.02	-
**8**	0.4-2.1	-	0.63	0.2-1.2	-	1.29	0.9-3.4	-	0.50	0.2-0.6	-	0
**9**	0.4-2.1	0.73	-	0.2-1.2	0.30	-	0.9-3.4	2.50	-	0.2-0.6	0.39	-
**10**	0.5-2.4	1.83	-	0.3-1.6	1.05	-	0.9-2.9	1.70	-	0.2-2.1	1.09	-
**11**	0.3-2.0	-	0.78	0.3-1.8	-	0.38	0.9-2.6	-	2.10	0.2-1.6	-	0.59
**12**	0.3-2.0	0.52	0.96	0.3-1.8	0.62	1.05	0.9-2.6	0.80	0.90	0.2-1.6	0.32	0.41
**13**	0.5-1.2	-	0.43	0.3-0.8	-	0.71	1.1-3.0	-	0.60	0.1-0.4	-	0.27
**14**	0.5-2.4	0.99	-	0.3-1.6	0.97	-	0.9-2.9	1.00	-	0.2-2.1	1.05	-
**15**	0.5-2.4	1.10	-	0.3-1.6	0.63	-	0.9-2.9	1.70	-	0.2-2.1	0.83	-
**16**	0.3-2.0	1.14	-	0.3-1.8	1.39	-	0.9-2.6	0.80	-	0.2-1.6	0.49	-
**17**	0.5-1.2	0.25	-	0.3-0.8	0.35	-	0.8-3.5	0.70	-	0.1-0.4	0.3	-
**18**	0.3-2.0	1.48	1.36	0.3-1.8	0.85	0.71	0.9-2.6	1.70	1.90	0.2-1.6	0.44	0.39
**19**	0.4-2.1	0.29	0.26	0.2-1.2	0.52	0.38	0.9-3.4	0.60	0.7	0.2-0.6	0.49	0.41
**20**	0.4-2.1	0.13	-	0.2-1.2	0.23	-	0.9-3.4	0.60	-	0.2-0.6	0.07	-
**21**	0.5-1.2	-	0.47	0.3-0.8	-	0.33	1.1-3.0	-	1.40	0.1-0.4	-	0.21
**22**	0.5-1.2	-	0.53	0.3-0.8	-	0.53	1.1-3.0	-	1.00	0.1-0.4	-	0.34
**23**	0.4-2.1	0.91	0.5	0.2-1.2	0.70	0.40	0.9-3.4	1.30	1.30	0.2-0.6	0.47	0.43
**24**	0.3-2.0	1.81	-	0.3-1.8	0.73	-	0.9-2.6	2.50	-	0.2-1.6	0.13	-
**25**	0.5-2.4	0.37	-	0.3-1.6	0.41	-	0.9-2.9	0.90	-	0.2-2.1	0.79	-
**26**	0.3-2.0	0.60	0.62	0.3-1.8	0.38	0.37	0.9-2.6	1.60	1.70	0.2-1.6	0.24	0.34
**27**	0.5-2.4	0.38	0.42	0.3-1.6	0.29	0.47	0.9-2.9	1.30	0.9	0.2-2.1	0.33	0.42
**28**	0.5-2.4	0.83	0.61	0.3-1.6	0.35	0.29	0.9-2.9	2.40	2.10	0.2-2.1	1.04	1.04
**29**	0.4-2.1	-	0.70	0.2-1.2	-	0.59	0.9-3.4	-	1.20	0.2-0.6	-	0.36

Data are available as presented; red color indicates values below normal range.

Supplemental Table 3: Antibodies for phenotypic analysis of B cells

Molecule	Clone	Fluorochrome	Manufacturer	Catalog #
**CD3**	UCHT1	BV510	Biolegend, San Diego, USA	344828
**CD14**	M5E2	BV510	Biolegend	301842
**CD56**	5.1H11	BV510	Biolegend	362534
**L/D**	–	Aqua (510)	Biolegend	423101
**CD19**	SJ25C1	BV711	Biolegend	363022
**CD27**	M-T271	BV421	Biolegend	356417
**IgD**	IA6-2	PE-CF594	Biolegend	348240
**RBD**	–	aFluor488	R&D Systems, Minneapolis, USA	AFG10500-020
**Full Spike**	–	Biotin	R&D Systems	BT10549-050
**Streptavidin**	–	APC	Biolegend	405207
**IgD**	IA6-2	PE-CF594	Biolegend	348240

Supplemental Table 4: Antibodies for functional analysis of T cells

Molecule	Clone	Fluorochrome	Manufacturer	Catalog #
**CD3**	SK7	PerCP/Cy5.5	Biolegend, San Diego, USA	344808
				563550
**CD4**	SK3	BUV395	BD, Heidelberg, Germany	47-0087-42
**CD8**	SK1	APCeFluor780	Thermo Fisher, Darmstadt, Germany	301842
**CD14**	M5E2	BV510	Biolegend	302242
**CD19**	H1B19	BV510	Biolegend	423101
**L/D**	–	Aqua (BV510)	Biolegend	304232
**CD45RO**	UCHL1	BV650	Biolegend	304834
**CD62L**	DREG-56	BV605	Biolegend	309810
**CD137**	4B4-1	APC	Biolegend	310824
**CD154**	24-31	BV421	Biolegend	502506
**IFNγ**	4S.B3	FITC	Biolegend	500326
**IL-2**	MQ1-17H12	PE-Cy7	Biolegend	500832
**IL-4**	MP4-25D2	PE-Dazzle594	Biolegend	502909

Supplemental Table 5: Statistics

Figure	HC$		SCTR §		Statistical test
	2nd	3rd	2nd	3rd	
**1A (left)**	18	11	22	17	One-way ANOVA with Sidak's multiple comparison test
**1A (right)**	-	-	14	14	paired t-test
**1B (left)**	18	11	23	17	Kruskal-Wallis with Dunn's multiple comparison test
**1B (right)**	-	-	14	14	Wilcoxon-matched pairs signed rank test
**1C (left)**	17	11	23	17	Fisher's exact test
**1C (middle)**	17	11	23	17	Kruskal-Wallis with Dunn's multiple comparison test
**1C (right)**	-	-	14	14	Wilcoxon-matched pairs signed rank test
**2A (left)**	17	12	21	15	Fisher's exact test
**2A (middle)**	15	10	17	13	Kruskal-Wallis with Dunn's multiple comparison test
**2A (right)**	-	-	9	9	Wilcoxon-matched pairs signed rank test
**2B (left)**	-	-	15	-	Mann-Whitney U test
**2C (left)**	15	10	17	13	One-way ANOVA with Sidak's multiple comparison test
**2C (right)**	15	10	17	13	Kruskal-Wallis with Dunn's multiple comparison test
**3A (left)**	18	11	22	19	Fisher's exact test
**3A (right)**	16	11	20	18	Kruskal-Wallis with Dunn's multiple comparison test
**3B (left)**	-	-	19	17	Simple linear regression
**3B (right)**	-	-	20	18	Simple linear regression
**3C (left)**	16	11	20	18	One-way ANOVA with Sidak's multiple comparison test
**3C (right)**	16	11	20	18	One-way ANOVA with Sidak's multiple comparison test
**3D (left)**	16	11	20	18	Kruskal-Wallis with Dunn's multiple comparison test
**3D (middle)**	16	11	20	18	One-way ANOVA with Sidak's multiple comparison test
**3D (right)**	16	11	20	18	One-way ANOVA with Sidak's multiple comparison test
**Table 1**					Mann-Whitney test, Fisher's exact test
**Supplemental Figure 1A (left)**	-	-	21	15	Simple linear regression
**Supplemental Figure 1A (right)**	-	-	23	17	Simple linear regression
**Supplemental Figure 1B**	18	11	22	17	Simple linear regression
**Supplemental Figure 1C**	17	11	23	17	Simple linear regression
**Supplemental Figure 2 B (left)**	-	-	17	13	Simple linear regression
**Supplemental Figure 2 B (right)**	-	-	16	12	Simple linear regression
**Supplemental Figure 3A**	15	10	17	13	Simple linear regression
**Supplemental Figure 3B**	15	10	17	13	Simple linear regression
**Supplemental Figure 3C**	15	10	17	13	Simple linear regression
**Supplemental Figure 3D**	15	10	17	13	Simple linear regression
**Supplemental Figure 3E**	15	10	17	13	Simple linear regression
**Supplemental Figure 5A**	16	11	20	18	Simple linear regression
**Supplemental Figure 5B**	16	11	20	18	Simple linear regression
**Supplemental Figure 5C**	16	10	20	18	Simple linear regression
**Supplemental Figure 5D**	16	11	20	18	Kruskal-Wallis with Dunn's multiple comparison test
**Supplemental Figure 5E**	16	11	20	18	One-way ANOVA with Sidak's multiple comparison test
**Supplemental Figure 5F (left)**	18	-	22	-	Fisher's exact test
**Supplemental Figure 5F (right)**	7	-	4	-	Mann-Whitney U test
**Supplemental Figure 6A (left)**	-	-	6/16#	5/12#	Unpaired t-test/ Mann-Whitney U test
**Supplemental Figure 6A (middle)**	-	-	6/17#	5/12#	Mann-Whitney U test
**Supplemental Figure 6A (right)**	-	-	6/17#	5/12#	Mann-Whitney U test
**Supplemental Figure 6B**	-	-	4/15#	3/10#	Mann-Whitney U test
**Supplemental Figure 6C**	-	-	5/15#	4/14#	Mann-Whitney U test

$/ § number of healthy individuals/SCTR included after 2nd and 3rd vaccination, respectively
number of SCTR included with and without GvHD, respectively
